# Factors which can influence elastic stable intramedullary nailing removal in healed bone cysts in children

**DOI:** 10.1038/s41598-024-61828-3

**Published:** 2024-05-15

**Authors:** Jiao Liu, Yuxi Su

**Affiliations:** Orthopedics Department, Children’s Hospital of Chongqing Medical University; Chongqing Key Laboratory of Pediatrics, Ministry of Education Key Laboratory of Child Development and Disorders; National Clinical Research Center for Child Health and Disorders; China International Science and Technology Cooperation Base of Child Development and Critical Disorders, Jiangxi Hospital Affiliated Children’s Hospital of Chongqing Medical University, Zhongshan 2Road 136#, Yuzhong District, Chongqing, 400014 People’s Republic of China

**Keywords:** Bone cyst, Children, Pathological fracture, Elastic stable intramedullary nailing removal, Factors, Paediatric research, Risk factors, Paediatrics

## Abstract

Elastic stable intramedullary nailing (ESIN) internal fixation is used clinically to treat pathological fractures of bone cysts in children. However, one of the most important complications was removal difficulty. In this study, we aim to analyse the factors which can influence ESIN removal in healed bone cysts in children. From April 2014 to November 2020, the clinical data of 49 children who underwent elastic stable intramedullary nail removal for pathological fractures of the bone cysts in our hospital were retrospectively analysed. The following data, including age, sex, pathological fracture site, with bone graft, number of ESINs, ESIN indwelling time, and extraosseous length of ESIN were collected, and univariate analysis and logistic regression analysis was performed. The frequency of difficulty in ESIN extraction was 44.90% (22/49). The univariate logistic regression analysis showed that age,ESIN indwelling time,with bone garft and extraosseous length of ESIN may be correlated with the difficulty in removing ESIN (*P* < 0.05), while sex, pathological fracture site, number of ESIN may not be correlated with the difficulty in removing ESIN (*P* > 0.05).The multivariate logistic regression analysis showed that the ESIN indwelling time was the independent influencing factor for difficulty in removing ESIN (*P* < 0.05). The factors influencing the ESIN removal in healed bone cysts in children include over 11.79 years old, the long indwelling time of the ESIN(over 10.5 months),with bone graft and short extraosseous length of ESIN(≤ 0.405 cm). These factors influencing ESIN removal in healed bone cysts in children should be considered.

## Introduction

Elastic stable intramedullary nailing (ESIN) is widely used to treat general fractures of the long bones in the limbs in children^1–3^. ESIN was recommended as a fixation material for paediatric shaft fractures of the forearms, humerus, femurs, and tibia. ESIN is minimally invasive (minimal operative stress), requires short hospital admission, simulates the bone's physiological healing process without requiring the fracture to be placed in a hard cast, the implant volume is low is economically feasible^4^, it has become widely accepted and recommended by the orthopaedic surgeon community. Although ESIN has substantive advantages in the treatment of fractures, there are complications, such as poor rotational function, skin irritation caused by the protruding nail end, delayed fracture healing, bone non-union, and removal difficulties^5–8^. Among these, ESIN removal is the most important, if neglected by the surgeon this usually causes iatrogenic injury. ESIN internal fixation is also used clinically to treat pathological fractures of bone cysts in children^9,10^. The implanted ESIN can continuously drain cyst fluid, relieving local pressure on the lesion, and improving venous blood flow to the lesions. It also acts as internal fixation, providing relative stability at the fracture site and promoting callus formation at the fracture site^11–13^. In this study, we retrospectively analysed the cases of 49 paediatric patients who underwent ESIN for pathological fractures of bone cysts at our hospital. We postulated that ESIN retention time, extraosseous length of ESIN, with bone graft, and age might be correlated with the ESIN removal. We chose bone cysts as a model to study ESIN removal based on our experience of difficult ESIN removal commonly occurring in these patients. The long retention of the ESIN together with the bone graft, pathological fractures, and different ages provided an ideal model for the analysis of factors. To the best of our knowledge, no study has focused on the factors that influence ESIN removal in children. This is the first study to investigate the factors for ESIN removal in children with pathological fracture of bone cysts. We evaluated the correlations between ESIN removal and ESIN retention time, bone graft use, extraosseous length of ESIN and patient age.

## Patients and methods

### Patients

Forty-nine patients treated with ESIN fixation for pathological bone cysts fractures at our hospital from April 2014 to November 2020 were retrospectively analysed, and clinical data were collected, including age, sex, pathological fracture site, whether bone grafting was performed, number of ESIN used, ESIN indwelling time, and extraosseous length of ESIN. Extraosseous length of ESIN was measured by two roentgenologist using plain radiographs, and the mean value was calculated. ESIN was divided into two groups according to whether it was difficult to remove the ESIN during the operation or not. An operating time of more than 40 min was considered a difficult removal. The operation time was recorded from the start of the skin incision to incision closing. The inclusion criterion was a pathological fracture of the bone cysts which confirmed by pathological biopsy, and ESIN fixation was performed in these patients. The exclusion criteria were other pathological or general fractures and surgical procedures where the internal fixator was not ESIN. This study was approved by the Ethics Committee of our hospital. Clinical data approval for publication was granted by patients’ guardians and data was anonymised.

### Surgical procedure

**Figure 1:**
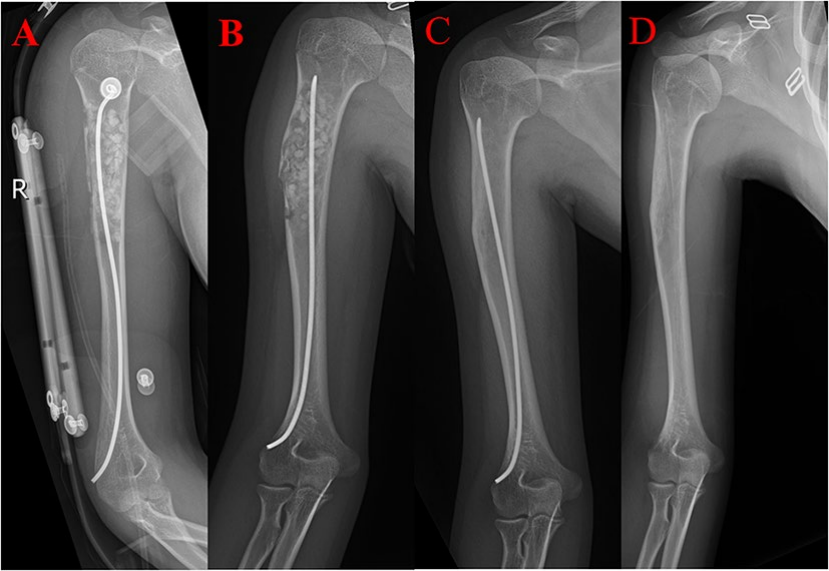
An 8-year-old female patient was diagnosed as pathological simple bone cyst (SBC) fracture of the right proximal humerus. (**A**) Patient underwent right humerus lesion removal, bone grafting and one elastic stable intramedullary nail fixation. (**B**) The SBS had a recurrence, the surgery was repeated 24 months postoperatively. (**C**) The SBC was totally healed. (**D**) The nail was removed, and the surgery time was 50 min another more 7 months.

After general anaesthesia, an incision was made according to the guidance of C-arm positioning ESIN (as far as possible at the original scar). The incision was about 1.5 cm long, the subcutaneous tissue was dissected layer by layer, exposing the the extraosseous part of ESIN. The ESIN was removed with nail forceps. If the ESIN cannot be removed using nail forceps, a special ESIN removal tool can be used to clamp the ESIN tail, and the nail can be successfully removed from the medullary cavity by rotating and changing the nail direction and repeatedly advancing and retreating the medullary cavity. If the ESIN cannot be removed, the bone window can be opened at the end with a bone knife, and a special tool for nail removal can be used to remove the ESIN smoothly. Certainly, for patients with ESIN that cannot be removed even with the use of specialised tools, ESIN can be driven retrogradely so that the ESIN head can penetrate the bone cortex, bite the bone cortex at the ESIN site, and finally the ESIN was removed. Finally, if the above methods (including window opening at the end of the nail, window opening at the nail, multiple advances, and retreat of the medullary cavity to remove the nail) remain unsuccessful in removing the ESIN, the surgeon would communicate with patients’ guardians for the ESIN to remain in the body. In which case the ESIN tail would be trimmed. Lastly, the C-arm confirmed the integrity of the ESIN removal. The incision was repeatedly rinsed with povidone and normal saline, and the skin was sutured subcutaneously with absorbable thread. Sterile dressing covers the incision (Fig. 1).

### Statistical analysis

SPSS (version 27.0; SPSS Inc., Chicago, IL, USA) was used for statistical analysis. Quantitative data were described as mean ± standard deviation or median (Q_1_, Q_3_). The t-test was used to compare the date that accord with the normal distribution. The Mann–Whitney test was used to compare the date that inconformity to the normal distribution. Categorical data were analysed by the chi-square test. Logistic regression analysiswas performed to identify influencing factors that might lead to difficulty in removing ESIN. *P* < 0.05 was considered statistically significant.

### Ethics approval and consent to participate

The study was approved by the ethics committee of Children’s Hospital of Chongqing Medical University. Informed and signed consent was taken from the parents or guardians before the operation. The study was conducted in accordance with relevant guidelines and regulations.

## Results

**Figure 2:**
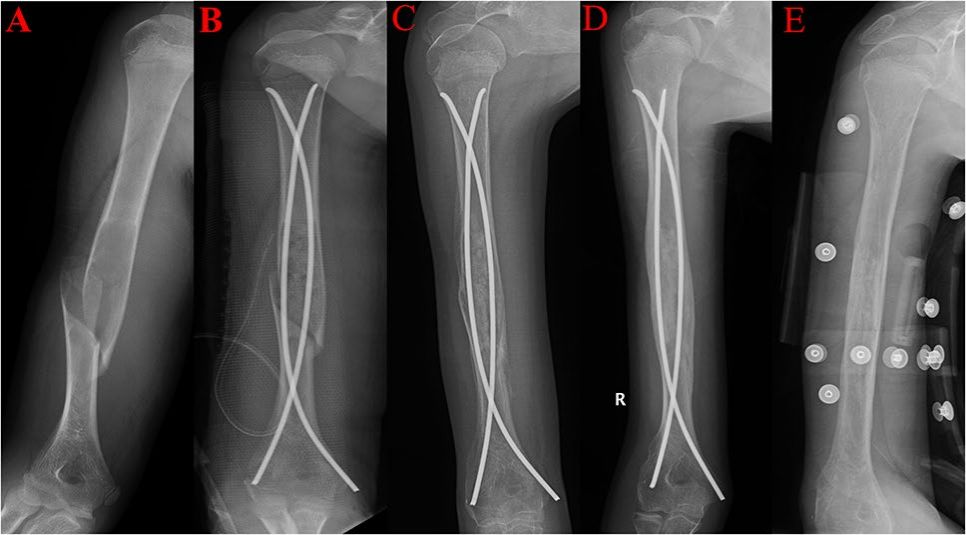
A 12-year-old male with pathological fracture of the right humerus. (**A**) The fracture before surgery on radiograph. (**B**) Right humerus lesion removal, bone grafting, and two elastic stable intramedullary nails fixation. (**C**) The fracture started to heal 2 months postoperatively. (**D**) The simple bone cyst and fracture was healed 7 months postoperatively. (**E**) Two nails were removed successfully, and the surgery duration was 10 min.

Forty-nine patients were included in this study, 35 were male and 14 were female, with an average age of 9.99 ± 3.35 years old, ranging from 2 to 18 years old. The mean indwelling time for ESIN was 4–31 months. Pathological fracture sites in descending order: Humerus; 31 patients (63.27%), Femurs; 15 patients (30.61%); the other place; 3(6.12%). Difficulty in removing ESIN was present in 22 cases, with fracture union (44.90%), including 14 males and 8 females, with an average age of 11.04 ± 3.49 years old, ranging from 4 to 18 years old, and an average indwelling time of ESIN of 14.00 months. The duration of indwelling ESIN ranged from 5 to 31 months. The most common pathological fracture site was the humerus (15 patients, 68.18%), followed by the femurs (6 patients, 27.27%) (Fig. 2). The clinical data of this study object are summarized in Tables 1 and 2.

**Table 1:** Continuous variables and comparative results.

Variables	Total (n = 49)	Difficult removal group (n = 22)	Easy removal group (n = 27)	Statistic	*P*
Age(years), Mean ± SD	9.99 ± 3.35	11.04 ± 3.49	9.14 ± 3.03	t = 2.05	0.046*
Indwelling time (months), M (Q_1_, Q_3_)	12.00 (8.00, 15.00)	14.00 (11.00, 22.50)	10.00 (7.50, 13.00)	Z = − 2.40	0.016*
Extraosseous length of ESIN (cm), M (Q_1_, Q_3_)	0.40 (0.28, 0.56)	0.34 (0.25, 0.50)	0.46 (0.35, 0.73)	Z = − 2.23	0.026*
Removing time (minutes), M (Q_1_, Q_3_)	35.00 (25.00, 60.00)	67.50 (50.00, 98.00)	28.00 (20.00, 35.00)	Z = − 5.98	< 0.001*

t: t-test, Z: Mann–Whitney test.

*SD* standard deviation, *M* median, *Q*_1_ 1st quartile, *Q*_3_ 3st quartile.

*P < 0.05 was considered significant.

**Table 2:** Categorical variables and comparative results.

Variables	Total (n = 49)	Difficult removal group (n = 22)	Easy removal group (n = 27)	Statistic	*P*
Sex, n(%)				Χ^2^ = 1.19	0.276
Female	14 (28.57)	8 (36.36)	6 (22.22)		
Male	35 (71.43)	14 (63.64)	21 (77.78)		
Bone graft, n(%)				χ^2^ = 4.23	0.040*
Yes	36 (73.47)	13 (59.09)	23 (85.19)		
No	13 (26.53)	9 (40.91)	4 (14.81)		
Fracture site, n(%)				–	0.824
Humerus	31 (63.27)	15 (68.18)	16 (59.26)		
Femurs	15 (30.61)	6 (27.27)	9 (33.33)		
Ulnar	1 (2.04)	0 (0.00)	1 (3.70)		
Tibia	1 (2.04)	0 (0.00)	1 (3.70)		
Fibula	1 (2.04)	1 (4.55)	0 (0.00)		
ESIN number, n(%)				χ^2^ = 0.53	0.468
1	16 (32.65)	6 (27.27)	10 (37.04)		
2	33 (67.35)	16 (72.73)	17 (62.96)		

*ESIN* elastic stable intramedullary nailing, *N* number.

χ^2^, Chi-square test, –, Fisher exact.

*P < 0.05 was considered significant.

There was significant difference in age, ESIN indwelling time, with bone graft, extraosseous length of ESIN between the difficult removal group and easy removal group (*P* < 0.05, Tables 1, 2).Children in the difficult removal group were older (11.04 ± 3.49) than that in the easy removal group (9.14 ± 3.03) when ESIN was removed (t = 2.05, *P* = 0.046). Indwelling time of ESIN in the difficult removal group were longer (14.00 (11.00, 22.50)) than that in the easy removal group (10.00 (7.50, 13.00)) (Z = − 2.40, *P* = 0.016). Extraosseous length of ESIN in the difficult removal group were shorter (0.34 (0.25, 0.50)) than that in the easy removal group (0.46 (0.35, 0.73)) (Z = − 2.23, *P* = 0.026).Whereas there was no significant difference in sex, fracture site, and number of ESINs used in two group. (*P* > 0.05, Table 2).

The univariate logistic regression analysis showed that age > 11.79 years (OR 6.9, 95% CI 1.78–26.71, *P* < 0.005), ESIN indwelling time > 10.5 months (OR 4.95, 95% CI 1.41–17.41, P < 0.013), with bone graft (OR 0.25, 95% CI 0.06–0.98, *P* < 0.046) and extraosseous length of ESIN ≤ 0.405 cm (OR 0.27, 95% CI 0.08–0.90, *P* < 0.033) were the factors that may lead to the difficult ESIN removal. The inclusion of above factors in multivariate logistic regression suggested that ESIN indwelling time is the independent influencing factor (Tables 3 and 4).

**Table 3:** Univariate logistic regression analysis affecting difficulty in removal of bone cyst pathological fractures after ESIN placement in children.

Variables	β	S.E	Z	*P*	OR (95% CI)
Sex					
Female					1.00 (Reference)
Male	− 0.69	0.64	− 1.08	0.279	0.50 (0.14–1.76)
Age(years)	0.188	0.098	1.928	0.054	1.21 (1–1.46)
Age To2c					
≤ 11.79	Ref.				
> 11.79	1.932	0.691	2.797	0.005*	6.9 (1.78–26.71)
Indwelling time (months)	0.15	0.06	2.51	0.012*	1.16 (1.03–1.30)
Indwelling time To2c					
≤ 10.5	Ref.				
> 10.5	1.598	0.642	2.490	0.013*	4.95 (1.41–17.41)
Bone graft					
Yes					1.00 (Reference)
No	− 1.38	0.69	− 1.99	0.046*	0.25 (0.06–0.98)
Extraosseous length of ESIN(cm)	− 2.698	1.326	− 2.036	0.042*	0.07 (0.01–0.9)
Extraosseous length of ESIN To2c					
≤ 0.405	Ref.				
> 0.405	− 1.293	0.607	− 2.130	0.033*	0.27 (0.08–0.9)
Fracture site	− 0.232	0.607	− 0.381	0.703	0.79 (0.24–2.61)

*OR* odds ratio, *CI* confidence interval.

*P < 0.05 was considered significant.

**Table 4:** Multivariate logistic regression analysis affecting difficulty in removal of bone cyst pathological fractures after ESIN placement in children.

Variables	β	S.E	Z	*P*	OR (95% CI)
Age To2c (> 11.79, ≤ 11.79)	0.594	0.663	0.896	0.370	1.81 (0.49–6.64)
Indwelling time To2c (> 10.5, ≤ 10.5)	1.517	0.674	2.250	0.024*	4.56 (1.22–17.1)
Extraosseous length of ESIN To2c (> 0.405, ≤ 0.405)	− 1.112	0.665	− 1.672	0.095	0.33 (0.09–1.21)

*OR* odds ratio, *CI* confidence interval.

*P < 0.05 was considered significant.

## Discussion

In this study, we first proved that age > 11.79 years, long indwelling time of ESIN(> 10.5 months), intraoperative bone grafting, and too short extraosseous length of ESIN (≤ 0.405 cm) were correlated with ESIN difficulty removal in children.

Bone cyst lesions lead to increased bone destruction and reduced bone firmness and are prone to pathological fracture^14^. Current clinical surgical methods for the treatment of pathological fractures of the SBC include steroid drug injection, autogenous red bone marrow injection transplantation, focal curettage, and bone grafting, combined with or without ESIN internal fixation^15^. Thus, we chose SBC as an ideal model for ESIN removal based on long-term indwelling, bone, graft, age, and sex etc.

In patients with difficult extraction, we found that ESIN had a longer median indwelling duration of 14 months in the difficult extraction group versus 10 months in the easy extraction group (*P* = 0.016). Due to the good capacity and quick recovery of fractures in children, it is very important for the successful removal of ESIN as early as possible. Fernandez et al.^16^ believed that the optimal interval for ESIN removal was 6 months post implantation. In a study by Emmanuel et al., the average time to remove ESIN was between 4 and 8.4 months^17^, both suggesting that ESIN was successfully removed within a year after initial internal fixation surgery^18^. Although there are two studies reported that the nails were removed at a mean of 30–40 months postoperatively^19,20^. If ESIN is retained in the body for too long, the nail body will be embedded in new bone, and finally ESIN will be integrated with the bone, resulting in difficulty of removing ESIN. Ardon et al. showed that the longer the internalisation time, the more difficult it is to remove the internal fixation^21^. Zhang believed that the later the removal of ESIN, the more difficult it is to remove the nail^22^. The results of this study are consistent with those of the above studies, suggesting that prolonged ESIN indwelling time is a risk factor for difficult removal after ESIN implantation in children with pathological fractures of the SBC.

Bone grafting provides a good microenvironment for bone formation and induces the growth of new bone, thus promoting the healing of pathological bone cyst fractures^23^. However, in the current literature, there are different opinions on whether bone grafting is necessary. Zhou et al. used either the combination of steroid hormones and ESIN or focal removal bone grafts and ESIN to treat children with simple bone cysts in two groups, and there was no statistically significant difference^12^. Kanellopoulos et al.^24^ treated nine patients with SBC by injecting autogenous iliac bone marrow mixed with decalcified bone matrix combined with ESIN; seven patients achieved Neer grade I union, and two patients achieved Neer grade II union. Erol et al. treated 34 patients with focal curettage, bone grafting, and ESIN fixation; 28 patients were completely healed (82%), 6 patients were partially healed (18%), and none of them experienced relapse^11^. In another study by Erol et al.^11^, 15 of 16 patients in the lesion curettage and bone graft plus ESIN treatment groups were completely healed, and only 1 patient was partially healed. Zhang et al.^22^, reported that all 18 patients with SBC achieved 100% complete or partial imaging healing after steroid hormone and ESIN treatment without recurrence or adverse reactions, and no children required a second cyst resection. According to the above studies, there is no reliable evidence that bone grafting can improve the cure rate of SBC compared to other treatment methods, and the results of this study suggest that intraoperative bone grafting is a risk factor for difficult removal after ESIN implantation for pathologic fractures of SBC in children. Our study demonstrated that bone grafting can be avoided as a complication of ESIN removal.

Our study also suggests that age > 11.79 years may significantly increase the probability of ESIN extraction difficulties. During the growth and development period of children, bone mineral density, bone size and other bone parameters show an increasing result with the increase of age^25,26^. After entering puberty, children begin their second growth and developmental peak, and their whole-body bones show linear growth. Therefore, older children have a higher bone density, which may be the reason for the difficulty in removing ESIN from older children.

In difficult extraction, we found that the extraosseous length of ESIN was shorter (median 0.34 cm in difficult extractions (median 0.46 cm) in easy extractions (*P* = 0.026). To successfully use the nail end in later stages, an appropriate extraosseous length of ESIN should be reserved during internal fixation of the ESIN. Luhmann et al.^27^ suggested that the ESIN tail should be exposed less than 2.5 cm from the cortex. Flynn and Parikh suggest that the nail should be left 1–2 cm outside the cortex^28,29^. Narayanan et al.^30^ believed that the length of the nail outside the cortical bone should be 1–1.5 cm. If the nail end is left too short, with long time growth, the nail end shear force will slowly embed it in the new bone or into the bone of the nail end. When inappropriately indwelled and placed near the cartilage or epiphyseal plate, the nail end tends to be covered by the cartilage, making it difficult to locate during ESIN removal. Luhmann et al.^27^ believed that too short nail end would lead to difficulty in removing ESIN. The research results of Simanovsky et al.^5^ show that short extraosseous length is the reason for the failure of later nail retrieval. Lieber et al.^31^ suggested that the duration of ESIN removal might be prolonged if the nail end is too short. The results of this study are consistent with those of the above studies, suggesting that a short extraosseous length of the ESIN (≤ 0.405 cm) is a risk factor for difficult removal after ESIN implantation for pathological fractures of the SBC in children (Fig. 2). Therefore, we recommend that the extraosseous length of the ESIN should be keeped at least 0.5cm, which may avoid the difficult extraction. Besides, we can add end caps to the nail ends, which can not only avoid the skin irritation, but also protecting the tip of ESIN from being encased by callus formation, thus tend to facilitate ESIN removal^32,33^.

This study had some limitations. First, this was a retrospective study with limited clinical data and possible factors considered. Other factors outside of those observed in this study may affect the difficulty of removing children with pathologic bone cyst fractures after ESIN implantation, such as the technical skills and experience of the surgeons, the surgical tools, etc. Therefore, we also need to constantly summarize the experience and strengthen the surgical skills of the ESIN insertion and removal and the fitting of end caps. Second, the length of the extraosseous length of the ESIN was measured in two dimensions directly on the X-radiographs, when in reality the extraosseous length of the ESIN has three dimensions. The use of fluoroscopy during surgery could prevent such inaccuracies. Third, there was no follow-up of recurrence rates after ESIN removal. At last, it needs the further study on the long-term effects of ESIN removal and non-removal on children, so as to develop a suitable treatment plan for Chinese children on whether ESIN should be removed. Last but not least, the potential flaw in the sample size may affect the veracity of this conclusion, which is illustrated by the large OR confidence interval, and a follow-up study with a large sample size is need to verify this conclusion.

## Conclusion

Age > 11.79 years, long indwelling time of ESIN(> 10.5 months), intraoperative bone grafting, and too short extraosseous length of ESIN (≤ 0.405 cm) all lead to difficulties in removing the ESIN,and the ESIN indwelling time is the independent influencing factor.

Therefore, these factors should be considered when using ESIN in the surgical treatment of pathological fractures of the SBC in children to reduce the occurrence of difficulty in removing ESIN.

## Data Availability

The datasets used and analyzed during the current study are available from the corresponding author on reasonable request.

## References

[1] Kamara A, Ji X, Liu T, Zhan Y, Li J, Wang E (2019). A comparative biomechanical study on different fixation techniques in the management of transverse metaphyseal-diaphyseal junction fractures of the distal humerus in children. Int. Orthop..

[2] Marengo L, Nasto LA, Michelis MB, Boero S (2018). Elastic stable intramedullary nailing (ESIN) in paediatric femur and tibia shaft fractures: Comparison between titanium and stainless steel nails. Injury.

[3] Zhang YT, Jin D, Niu J, Li ZJ, Fu S, Zou ZL (2016). A meta-analysis of external fixation and flexible intramedullary nails for femoral fractures in children. Acta Orthop. Belg..

[4] Cosma D, Vasilescu DE (2014). Elastic stable intramedullary nailing for fractures in children - specific applications. Clujul. Med..

[5] Simanovsky N, Tair MA, Simanovsky N, Porat S (2006). Removal of flexible titanium nails in children. J. Pediatr. Orthop..

[6] Sun XS, Wang B, Wang F (2018). Complications of 2 133 cases of pediatric long bone fracture undergoing elastic stable intramedullary nailing in a single medical center. Zhonghua Wai Ke Za Zhi.

[7] Pogorelić Z, Vodopić T, Jukić M, Furlan D (2019). Elastic stable intramedullary nailing for treatment of pediatric femoral fractures; A 15-year single centre experience. Bull. Emerg. Trauma.

[8] Bajelidze G, Beruashvili Z, Bajelidze L, Zimlitski M (2019). Complications of treatment by titanium elastic intramedullary nails in children with femoral shaft fractures. Georgian Med. News.

[9] Zhang KX, Chai W, Zhao JJ, Deng JH, Peng Z, Chen JY (2021). Comparison of three treatment methods for simple bone cyst in children. BMC Musculoskelet. Disord..

[10] Wang X, Han J, Li Y, Liu Y, Luo J (2021). Comparative efficacy and safety profile for the treatment of humeral bone cysts in children: Curettage and mixed bone grafting either with or without elastic intramedullary nailing. J. Orthop. Surg. Res..

[11] Erol B, Onay T, Çalışkan E, Aydemir AN, Topkar OM (2015). Treatment of pathological fractures due to simple bone cysts by extended curettage grafting and intramedullary decompression. Acta Orthop. Traumatol. Turc.

[12] Zhou J, Ning S, Su Y, Liu C (2021). Elastic intramedullary nailing combined with methylprednisolone acetate injection for treatment of unicameral bone cysts in children: A retrospective study. J. Child Orthop..

[13] Erol B, Onay T, Topkar OM, Tokyay A, Aydemir AN, Okay E (2017). A comparative study for the treatment of simple bone cysts of the humerus: Open curettage and bone grafting either without instrumentation or with intramedullary nailing. J. Pediatr. Orthop. B..

[14] Pavone V, Caff G, Di Silvestri C, Avondo S, Sessa G (2014). Steroid injections in the treatment of humeral unicameral bone cysts: Long-term follow-up and review of the literature. Eur. J. Orthop. Surg. Traumatol..

[15] Kadhim M, Thacker M, Kadhim A, Holmes L (2014). Treatment of unicameral bone cyst: Systematic review and meta analysis. J. Child Orthop..

[16] Fernandez FF, Langendörfer M, Wirth T, Eberhardt O (2010). Failures and complications in intramedullary nailing of children’s forearm fractures. J. Child Orthop..

[17] Gibon E, Béranger JS, Bachy M, Delpont M, Kabbaj R, Vialle R (2015). Influence of the bending of the tip of elastic stable intramedullary nails on removal and associated complications in pediatric both bone forearm fractures: A pilot study. Int. J. Surg..

[18] Lascombes P, Prevot J, Ligier JN, Metaizeau JP, Poncelet T (1990). Elastic stable intramedullary nailing in forearm shaft fractures in children: 85 cases. J. Pediatr. Orthop..

[19] Roposch A, Saraph V, Linhart WE (2000). Flexible intramedullary nailing for the treatment of unicameral bone cysts in long bones. J. Bone Joint Surg. Am..

[20] Chen CF (1991). Effects of atrial natriuretic factor on renal function in chronic hypoxic rats. J. Formos Med. Assoc..

[21] Ardon H, Van Calenbergh F, Van Raemdonck D (2009). Oesophageal perforation after anterior cervical surgery: Management in four patients. Acta Neurochir. (Wien).

[22] Zhang P, Zhu N, Du L, Zheng J, Hu S, Xu B (2020). Treatment of simple bone cysts of the humerus by intramedullary nailing and steroid injection. BMC Musculoskelet. Disord..

[23] Hagmann S, Eichhorn F, Moradi B (2011). Mid- and long-term clinical results of surgical therapy in unicameral bone cysts. BMC Musculoskelet. Disord..

[24] Kanellopoulos AD, Mavrogenis AF, Papagelopoulos PJ, Soucacos PN (2007). Elastic intramedullary nailing and DBM-bone marrow injection for the treatment of simple bone cysts. World J. Surg. Oncol..

[25] Bonjour JP, Chevalley T, Ammann P, Slosman D, Rizzoli R (2001). Gain in bone mineral mass in prepubertal girls 3.5 years after discontinuation of calcium supplementation: A follow-up study. Lancet.

[26] Wetzsteon RJ, Petit MA, Macdonald HM, Hughes JM, Beck TJ, McKay HA (2008). Bone structure and volumetric BMD in overweight children: A longitudinal study. J. Bone Miner Res..

[27] Luhmann SJ, Schootman M, Schoenecker PL, Dobbs MB, Gordon JE (2003). Complications of titanium elastic nails for pediatric femoral shaft fractures. J. Pediatr. Orthop..

[28] Flynn JM, Hresko T, Reynolds RA, Blasier RD, Davidson R, Kasser J (2001). Titanium elastic nails for pediatric femur fractures: A multicenter study of early results with analysis of complications. J. Pediatr. Orthop..

[29] Parikh SN, Jain VV, Denning J (2012). Complications of elastic stable intramedullary nailing in pediatric fracture management: AAOS exhibit selection. J. Bone Joint Surg. Am..

[30] Narayanan UG, Hyman JE, Wainwright AM, Rang M, Alman BA (2004). Complications of elastic stable intramedullary nail fixation of pediatric femoral fractures, and how to avoid them. J. Pediatr. Orthop..

[31] Lieber J, Dietzel M, Scherer S, Schäfer JF, Kirschner HJ, Fuchs J (2022). Implant removal associated complications after ESIN osteosynthesis in pediatric fractures. Eur. J. Trauma Emerg. Surg..

[32] Nectoux E, Giacomelli MC, Karger C, Gicquel P, Clavert JM (2008). Use of end caps in elastic stable intramedullary nailing of femoral and tibial unstable fractures in children: preliminary results in 11 fractures. J. Child. Orthop..

[33] Slongo T, Audigé L, Hunter JB, Berger SM (2011). Clinical evaluation of end caps in elastic stable intramedullary nailing of femoral and tibial shaft fractures in children. Eur. J. Trauma Emerg. Surg..

